# Protocol for detection of resistance gene expression induced by mechanical wounding in tomato leaves

**DOI:** 10.1016/j.xpro.2026.104945

**Published:** 2026-11-05

**Authors:** Qinyang Wang, Lei Deng, Ming Zhou, Guoliang Yuan, Chuanyou Li, Changbao Li

**Affiliations:** 1 Key Laboratory of Biology and Genetic Improvement of Horticultural Crops (North China), Ministry of Agriculture, Beijing Institute of Vegetable Science, Beijing Academy of Agriculture and Forestry Sciences, Beijing 100097, China; 2 Taishan Academy of Tomato Innovation, Shandong Agricultural University, Tai^’^an 271018, China; 3 College of Life Sciences, Shandong Agricultural University, Tai^’^an 271018, China

**Keywords:** Plant sciences, Molecular Biology, Gene Expression

## Abstract

Systemic resistance is vital to tomato defense research, but quantifying its response poses major challenges. Here, we present a protocol for detecting the expression level of resistance genes induced by mechanical wounding in tomato leaves. We describe steps for seedling cultivation, wounding treatment, RNA extraction, cDNA reverse transcription, and qPCR detection. This protocol has broad potential applications in studying the systemic resistance response.

For complete details on the use and execution of this protocol, please refer to Zhou et al.^1^

## Highlights

• Integrated pipeline for wound-triggered resistance assays in tomato leaves
• Streamlined RNA extraction for transcript profiling of tomato leaves
• Step-by-step reverse transcription for parallel multi-leaf sample cDNA synthesis
• RT-qPCR workflow to quantify defense gene expression in systemic leaves

## Abstract (editor highlights)

Publisher’s note: Undertaking any experimental protocol requires adherence to local institutional guidelines for laboratory safety and ethics.

## Graphical abstract

**Figure:**
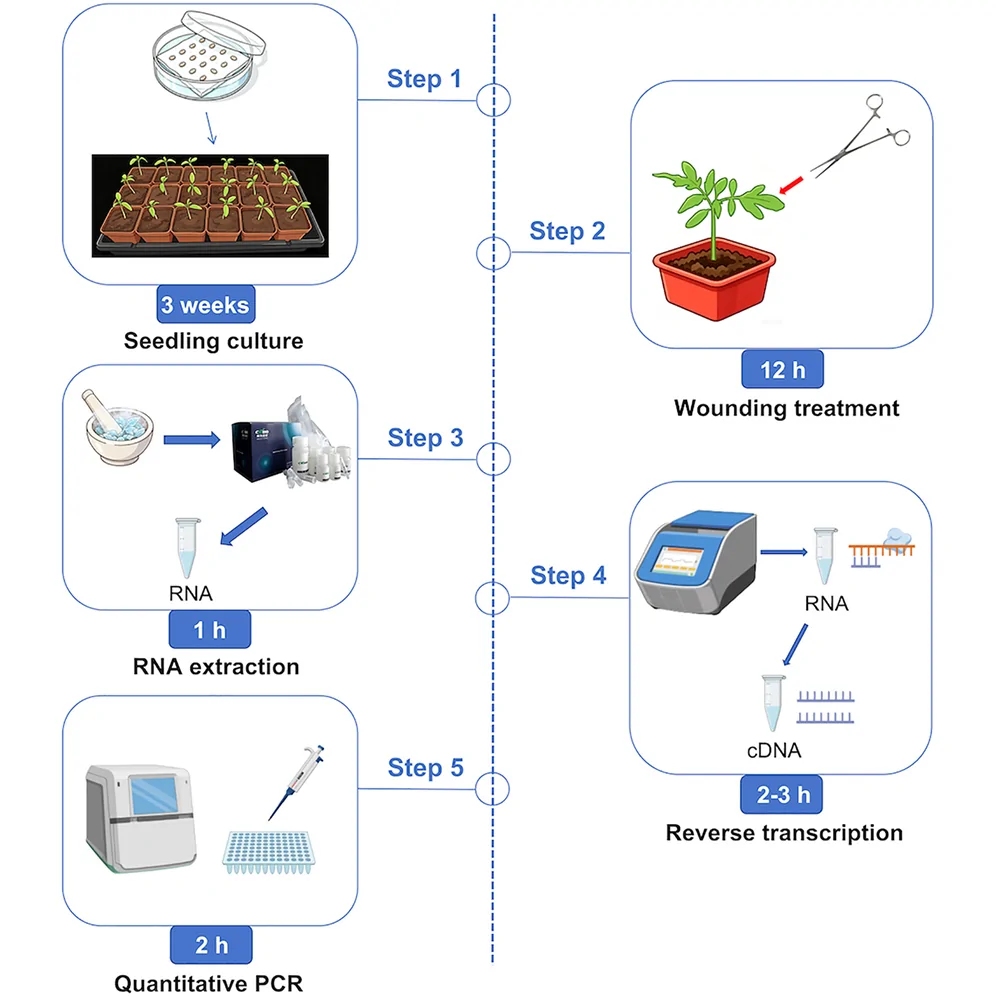

## Before you begin

Tomato (*Solanum lycopersicum*) is a classic model plant for studying plant defense response. When tomato leaves are subjected to chewing insects or mechanical wounding, they accumulate large amounts of defensive compounds (such as proteinase inhibitors) both at local wounded leaves and distal systemic leaves, indicating that plants possess systemic resistance response.^2^^,^^3^^,^^4^

Measuring wound-induced resistance gene transcripts precisely is key to uncovering tomato’s molecular responses to mechanical wounding.^5^^,^^6^ This detection also supplies theory and techniques to breed stress-tolerant tomato lines, improve field management, and lessen yield loss from mechanical wounding. Yet the assay faces several technical hurdles. Variations in wounding strength, position or time cause inconsistent gene induction. Unspecific quantification methods may further trigger false positive or negative signals.

This protocol incorporates a number of standard operating procedures to overcome these technical difficulties. In order to accomplish precise transcript quantification, it encompasses the precise seedling culture method, standard mechanical wounding treatment, RNA extraction, and specific quantitative PCR.

The protocol is simple to operate and highly repetitive, with stable and reliable data. It is applicable to various research directions: functional analysis of systemic resistance response genes, screening of core regulatory factors in systemic resistance response pathway, and selecting tomato genotypes with strong wound tolerance. With minor modifications, this protocol works for other *solanaceous* crops such as pepper and potato. This protocol supports research on plant stress physiology, molecular pathology and crop resistance breeding.

### Innovation

This protocol develops an integrated quantitative pipeline for profiling defense gene expression in mechanically wounded tomato leaves. The protocol unifies standardized mechanical wounding treatment, RNA extraction, reverse transcription and qPCR analysis to simplify overall experimental operations. Optimizations applied to all experimental steps further improve the reliability and reproducibility of subsequent molecular detection results. The method is easy to operate and suitable for experiments. It can support gene screening, functional validation and wounding response assays under varied environments. The tool promotes systematic studies focusing on tomato resistance response activated by mechanical wounding.

## Key resources table

**Table:** 

REAGENT or RESOURCE	SOURCE	IDENTIFIER
**Critical commercial assays**		

Ultrapure RNA Kit	CWBIO	Cat#: CW0581S
PrimeScript™ RT reagent Kit	TaKaRa	Cat#: RR047A
qPCR reagent Kit	KAPA	Cat#: KK4601

**Experimental models: Organisms/strains**		

Tomato cultivar: AC	Zhou et al.^1^	N/A

**Oligonucleotides**		

*ACTIN2* (SGN: Solyc11g005330) qPCR primer pair (F:TTGCTGACCGTATGAGCAAG; R:GGACAATGGATGGACCAGAC)	This paper	N/A
*PI-II* (GenBank: K03291.1) qPCR primer pair (F:AATTATCCATCATGGCTGTTCAC; R:CCTTTTTTGGATCAGATTCTCCTT)	This paper	N/A

**Software and algorithms**		

ImageJ v1.54f Generic Diagramming Platform (BioGDP)	Schneider et al.^7^; Jiang et al.^8^	https://imagej.nih.gov/ij/; https://biogdp.com

**Other**		

Real-Time PCR System Spectrophotometer LightCycler Multiwell Plate 96 Germination paper Straight hemostatic forceps Straight tweezers	Roche Thermo Roche EASYBIO Biosharp BKMAM	LightCycler 480 NanoDrop Cat#:04729692001 Cat#:BE6654 Cat#: BS-HF-S-140 BK-DF16-ST

## Materials and equipment

**CRITICAL:** Trizol reagent contains toxic and corrosive components. Avoid skin, eye and respiratory contact. Perform all operations in a fume hood with nitrile gloves and safety goggles.

**CRITICAL:** Chloroform is volatile, toxic and irritant. It may damage respiratory tract and eyes. Always use in a well-ventilated fume hood and wear protective gloves and goggles.

## Step-by-step method details

### Tomato seedling preparation and wounding treatment

**Timing: 3 weeks**

This section covers standardized tomato seed germination, seedling cultivation and uniform mechanical leaf wounding. Consistent seedling age and identical wounding intensity are critical to minimize variation in wound-induced defense gene expression.

1. Tomato seed germination:
  a. Select culture dishes with 9 cm diameter.
  b. Place the seed germination paper at the bottom of the culture dish.
  c. Surface-sterilize tomato seeds prior to germination to minimize microbial contamination.
    i. Rinse seeds with 75% ethanol for 2 min.
    ii. Incubate seeds in 5% sodium hypochlorite solution for 10 min with gentle shaking.
    iii. Wash seeds 6–7 times with sterile distilled water to remove residual disinfectant.
  d. Spread the tomato seeds evenly on the germination paper.
  e. Add distilled water to the culture dish to fully moisten the germination paper. Ensure seeds are in full contact with water, but not submerged under the water.
  f. Culture the seeds in the dark for 2 to 3 days until the radicle just begins to emerge.

***Note:*** Germination paper is an ISTA compliant, chemically inert, highly absorbent cellulose blotting paper designed for seed germination assays, free of bleaching agents or toxic additives that may disturb seed physiology or subsequent gene-expression measurements.

**CRITICAL:** Surface sterilization is required to minimize microbial contamination. Without sterilization, fungal or bacterial contamination may occur during germination, which can induce plant defense responses and alter defense-related gene expression, confounding downstream qPCR analysis.

**CRITICAL:** Sow germinated seeds before cotyledons turn fully green. 16 cm straight tweezers are used to sow germinated seeds. Otherwise, when the seeds are sown in the tray, they are likely to be subjected to stress.

2. Seedling transplantation and cultivation:
  a. Fill small square plastic pots (7 cm side length; round pots of equivalent volume are also acceptable) with the prepared growing substrate.
  b. Move the tomato seeds whose radicles have broken through the seed coat into the seedling tray.
  c. Wait for the tomato seedlings to grow into a two leaf, one heart state (Figure 1).

**Figure 1:**
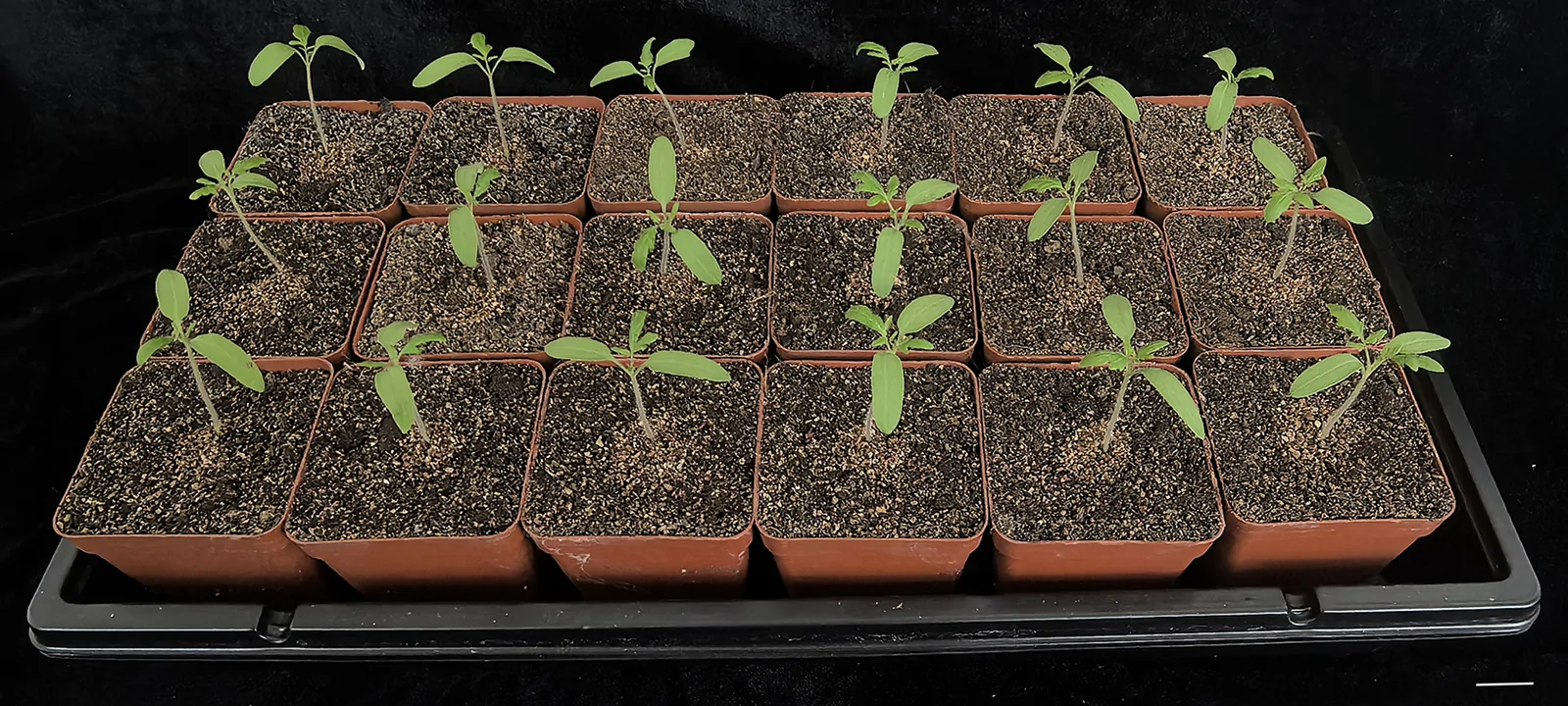
Representative image of tomato seedling cultivation The tomato seedlings were planted in small square plastic pots. Each genotype contains at least 8 seedlings to ensure the statistical reliability of the detection of the expression level of resistance genes induced by mechanical damage. Materials of different genotypes should be planted in the same tray to ensure that the growth conditions of the plants are the same. Scale bars = 2 cm.

***Note:*** The substrate is prepared by mixing vermiculite and peat in a 1:1 volume ratio.

**CRITICAL:** During the growth period of tomato seedlings, it is necessary to control the water supply to avoid stress caused by excessive watering. Grow seedlings in a growth chamber under controlled conditions (16 h of light at 26°C and 8 h of dark at 18°C with 60% relative humidity, light intensity: 180–220 μmol m^−2^ s^−1^). No additional fertilizer is required at this stage, since the substrate provides adequate nutrients.

3. Mechanical wounding treatment of tomato leaves:
  a. Select healthy tomato seedlings at the two leaf, one heart stage.
  b. Sterilize 14 cm straight hemostatic forceps with 75% ethanol and air dry completely before use.
  c. Use 14 cm straight hemostatic forceps to wound the lamina of the first true leaf by one single clamp with consistent pressure; this treatment produces visible leaf drooping (Figure 2).

**Figure 2:**
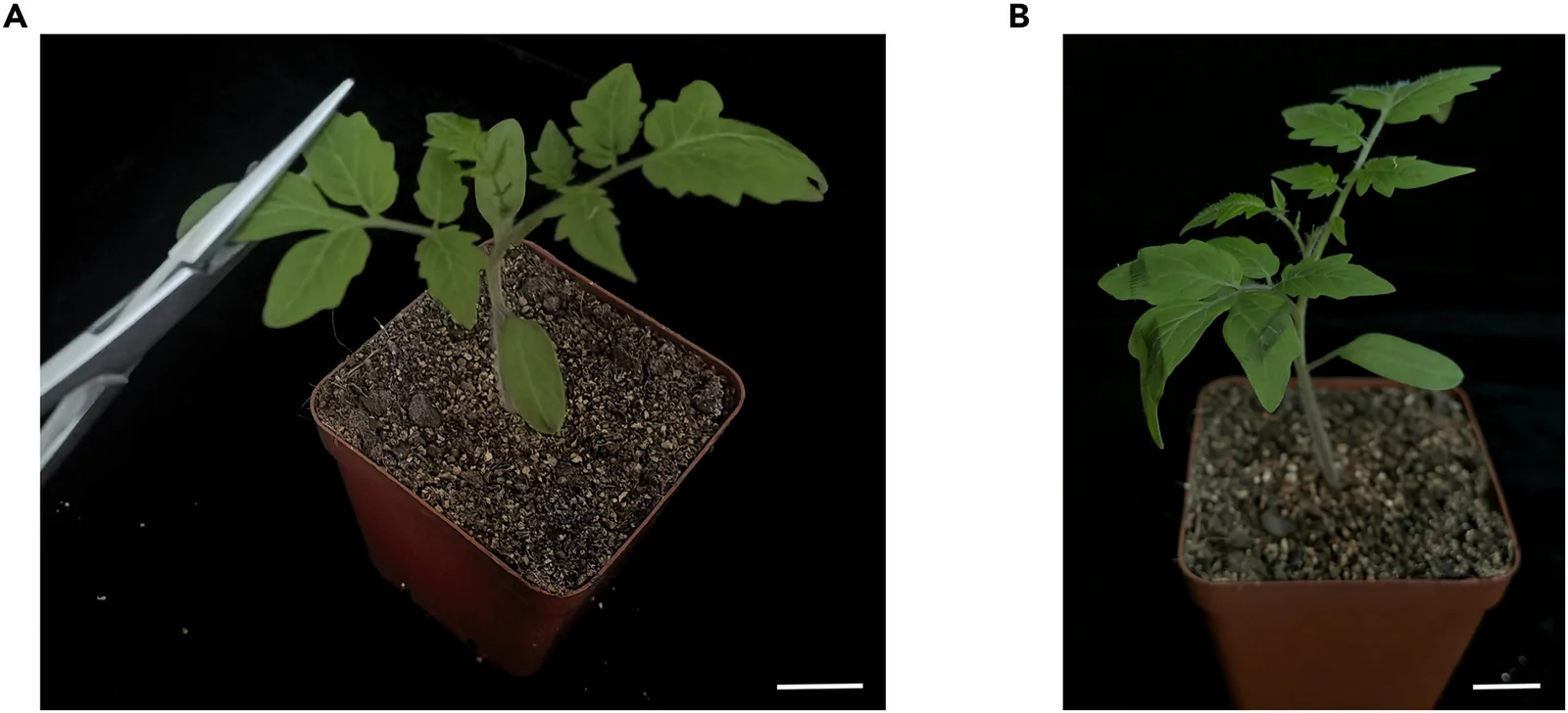
Schematic presentation of mechanical wounding treatment on tomato seedlings (A) Wait until the tomato seedlings reach the two-leaf-one-heart stage, then use hemostatic forceps to mechanically wound the first true leaf. (B) The wounded leaf tissues exhibited obvious downward wilting at the defined sampling time. Scale bars = 2 cm.
  d. Number and label treated seedlings, and set up an untreated control group consisting of independent seedlings grown in separate pots under identical growth conditions.
  e. Harvest three separate samples at 12 h post-treatment, followed by rapid snap-freezing in liquid nitrogen:
    i. The wounded leaflet of the first true leaf (only the wounded leaflet, not the whole trifoliate leaf, is harvested for this local sample).
    ii. The undamaged second true leaf from the same seedling for systemic response analysis.
    iii. Undamaged leaves from independent untreated seedlings (control).

***Note:*** Ensure the wounded area on the leaf blade faces vertically downward after mechanical wounding.

***Note:*** Separate sampling should be performed for mechanically wounded leaves and undamaged second true leaves (systemic) collected from the same wounded seedlings, as well as untreated control leaves harvested from independent untreated tomato seedlings (control).

**CRITICAL:** Sterilize the hemostatic forceps between every individual seedling and between different genotypes to avoid cross-contamination of plant sap.

4. Leaf sample storage:
  a. Place leaves frozen in liquid N2 directly into a −80°C freezer right after collection.
  b. Clearly label each sample for tissue type (control, wounded, systemic leaf), treatment time (12 h after wounding) and sample serial number.

**CRITICAL:** Repeated freezing and thawing events will degrade RNA and other biomolecules; minimize changes in storage temperature for stored samples.

**CRITICAL:** Perform RNA extraction within 3 months after sampling. Long-term storage may cause RNA degradation and compromise the reliability of qPCR results.

### Total RNA extraction from tomato leaves

**Timing: 2–3 h**

This step details total RNA isolation from control, wounded and systemic tomato leaf tissue via column-based purification. Intact, high-purity RNA is essential to obtain reliable cDNA for subsequent quantitative transcription analysis.

5. Grind leaf samples:
  a. Retrieve frozen leaf tissue from the −80°C freezer and put this into a cold mortar.
  b. Continue adding liquid nitrogen while mortar grinding to ensure tissue is completely frozen.
  c. Mix powdered tissue to a pre-cooled 1.5 mL centrifuge tube, with the amount of powdered tissue up to the 500 μL line.

***Note:*** Grind tissue completely to fine powder without any visible leaf pieces. Not enough grinding results in a lower RNA final quality.

***Note:*** The powdered ground tissue can be divided into two tubes. One tube will be held in as a reserve.

***Note:*** Ground leaf powder must remain completely frozen throughout the procedure. Do not allow the tissue to thaw at any point before adding Trizol reagent, as thawing will cause RNA degradation.

6. RNA Extraction:
  a. Add 1 mL Trizol reagent to the tissue powder, mix well, and leave the mixture at room temperature for 5 min.
  b. Transfer the mixed solution into the adsorption column placed in the collection tube in two portions. Centrifuge at 4°C and 13500 × *g* for 20 s, then discard the solution in the collection tube.
  c. Add 700 μL of Buffer RW1 to the adsorption column, centrifuge at 4°C at 13500 × *g* for 20 s, and discard the solution in the collection tube.
  d. Add 500 μL of Buffer RW2 reagent to the adsorption column. Centrifuge at 4°C for 13500 × *g* for 20 s. Discard the solution in the collection tube. Repeat this step once.
  e. Centrifuge at 13500 × *g* for 2 min at 4°C, then let the adsorption column dry completely.
  f. Place the adsorption column in a new 1.5 mL RNase-free centrifuge tube. Add 30 μL of RNase-free water to the center of the column and let it stand for 1 min.
  g. Centrifuge at 13500 × *g* for 1 min at 4°C to collect RNA solution, then quantify RNA concentration via spectrophotometry.
  h. Detect RNA concentration and purity via NanoDrop spectrophotometer (Figure 3).

**Figure 3:**
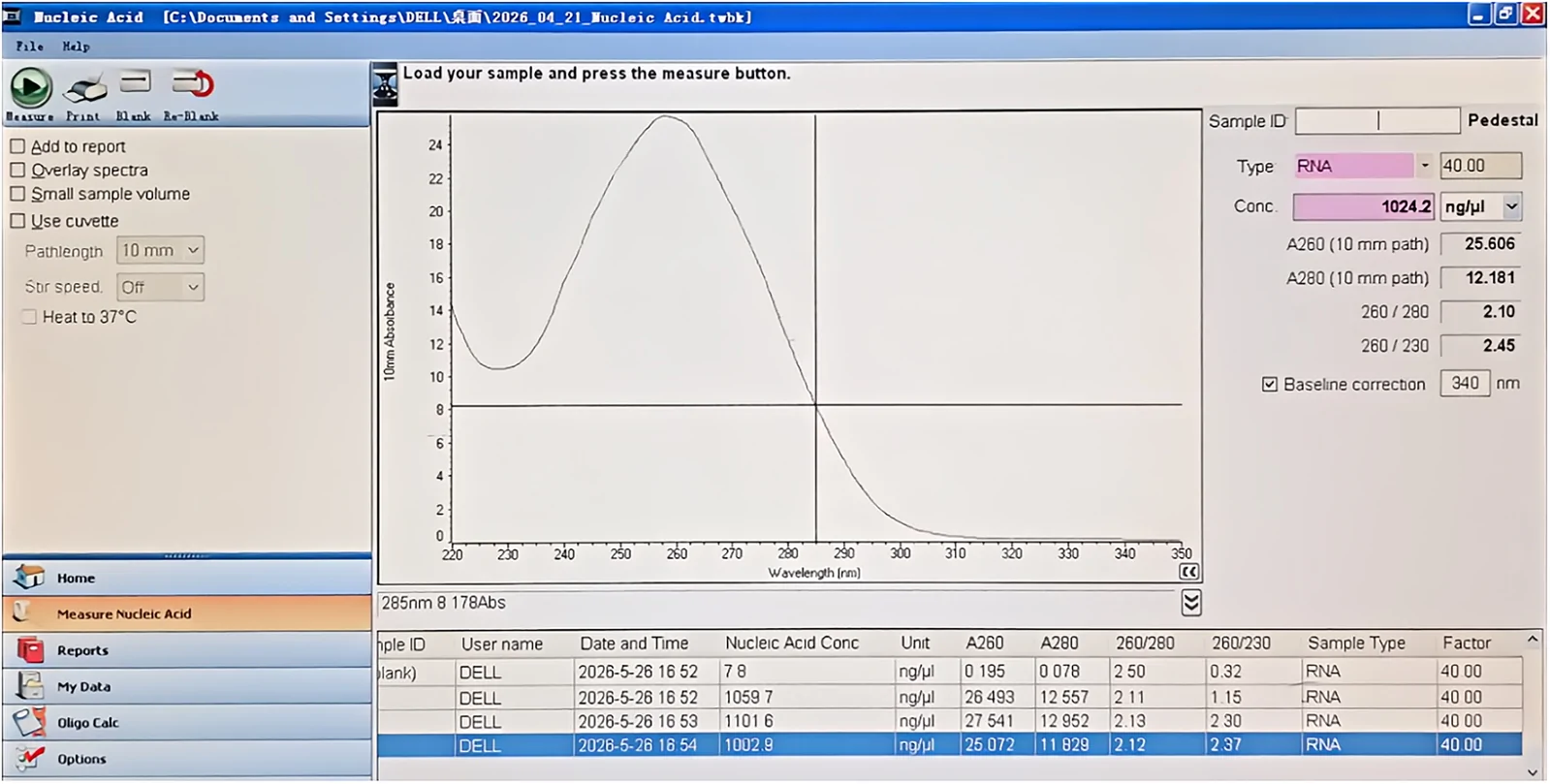
Quality and concentration detection of total RNA extracted from tomato leaves RNA quality and concentration were analyzed using a spectrophotometer. The absorbance at 260 nm was measured for concentration calculation, while the A260/A280 and A260/A230 ratios were used to assess RNA purity.
  i. Validate RNA integrity by agarose gel electrophoresis before reverse transcription.
  j. Finally, store the RNA in a −80°C refrigerator.

***Note:*** The extracted RNA samples should be avoided from undergoing repeated freezing and thawing, as this will adversely affect the quality of the RNA.

***Note:*** Alternative column-based RNA extraction kits are also compatible, as long as they can produce intact and high-purity total RNA from plant leaf tissue.

### Reverse transcription for cDNA synthesis

**Timing: 1 h**

Here we outline DNase treatment and first-strand cDNA synthesis from purified leaf RNA. Complete genomic DNA removal ensures accurate quantification of target wound-responsive transcripts in downstream qPCR.

7. Reverse Transcription:
  a. Prepare all reaction reagents on ice to avoid RNA degradation and reagent inactivation (Figure 4).

**Figure 4:**
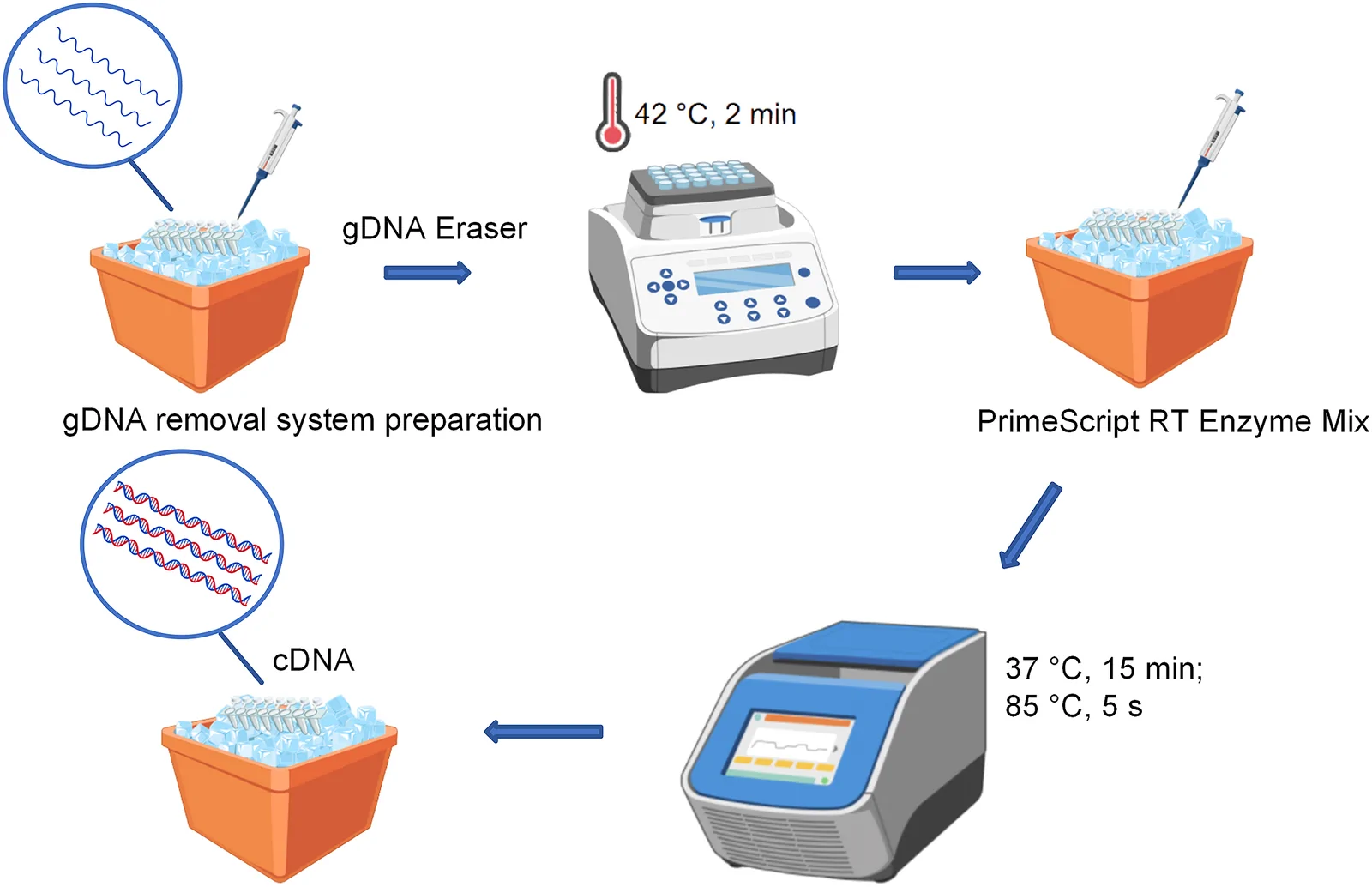
Schematic reverse transcription workflow of RNA RNA extracted from tomato leaves was mixed with gDNA Eraser on ice, incubated at 42 °C for 2 min to remove genomic DNA. PrimeScript RT Enzyme Mix was added under ice-cold RNase-free condition, then thermal cycler programmed incubation generated first-strand cDNA, which was ready for qPCR analysis of wound-responsive gene expression. (Created with BioGDP).
  b. Add the following reagents to a sterile, RNase-free centrifuge tube in sequence:

**Table:** 

Reagent	Amount
2 μl 5X gDNA Eraser Buffer	2 μL
gDNA Eraser	1 μL
total RNA	1 μg
ddH_2_O	Adjust to 10 μL
  c. Incubate at 42°C for 2 min, then place the tube on ice temporarily.
  d. Add 10 μL of RT master mix into the above 10 μL gDNA-removed solution, mix gently and spin:

**Table:** 

Reagent	Amount
5×PrimeScript Buffer 2	4 μL
PrimeScript RT Enzyme Mix I	1 μL
RT Primer Mix	1 μL
ddH_2_O	4 μL
  e. Incubate at 37°C for 15 min, followed by 85°C for 5 s to terminate the reaction. Store cDNA on ice or at −80°C for long-term preservation.
    ***Note:*** Genomic DNA is eliminated via 2 min incubation at 42°C using gDNA Eraser possessing potent DNA-degrading activity.
    ***Note:*** Alternative cDNA synthesis kits can also be used according to researchers’ preference, provided they can generate high-quality first-strand cDNA suitable for subsequent qPCR analysis.
    ***Note:*** When multiple reactions need to be carried out simultaneously, the mixed solutions of various reagents (Master Mix; which include RNase Free ddH_2_O, Buffer, enzymes, etc.) should be prepared first, and then aliquoted into each reaction tube. This will ensure a more accurate volume of the reagents taken, reduce the loss of reagents, and avoid repeatedly taking the same reagent. At the same time, it can also minimize the errors caused by experimental operations or between experiments.

### Quantitative real-time PCR and data analysis

**Timing: 2 h**

This workflow describes SYBR Green-based qPCR setup and statistical normalization of defense gene transcripts. Melting curve validation and statistical testing are required to confirm specific, reproducible gene expression results.

8. Quantitative RT-PCR setup:
  a. Use the Kapa biosystem for qPCR analysis. The 20 μL reaction system includes:

**Table:** 

Reagent	Amount
cDNA	2 μL
SYBR Fast qPCR Kit	10 μL
RT-F primer (0.25 μM)	0.5 μL
RT-R primer (0.25 μM)	0.5 μL
ddH_2_O	Adjust to 20 μL
  b. Use the Roche LightCycler 480 real-time PCR system with the following cycling conditions (Figure 5):

**Table:** 

Steps	Temperature	Time	Cycles
Initial Denaturation	95°C	30 s	1
Denaturation	95°C	5 s	40 cycles
Annealing	59°C	30 s

**Figure 5:**
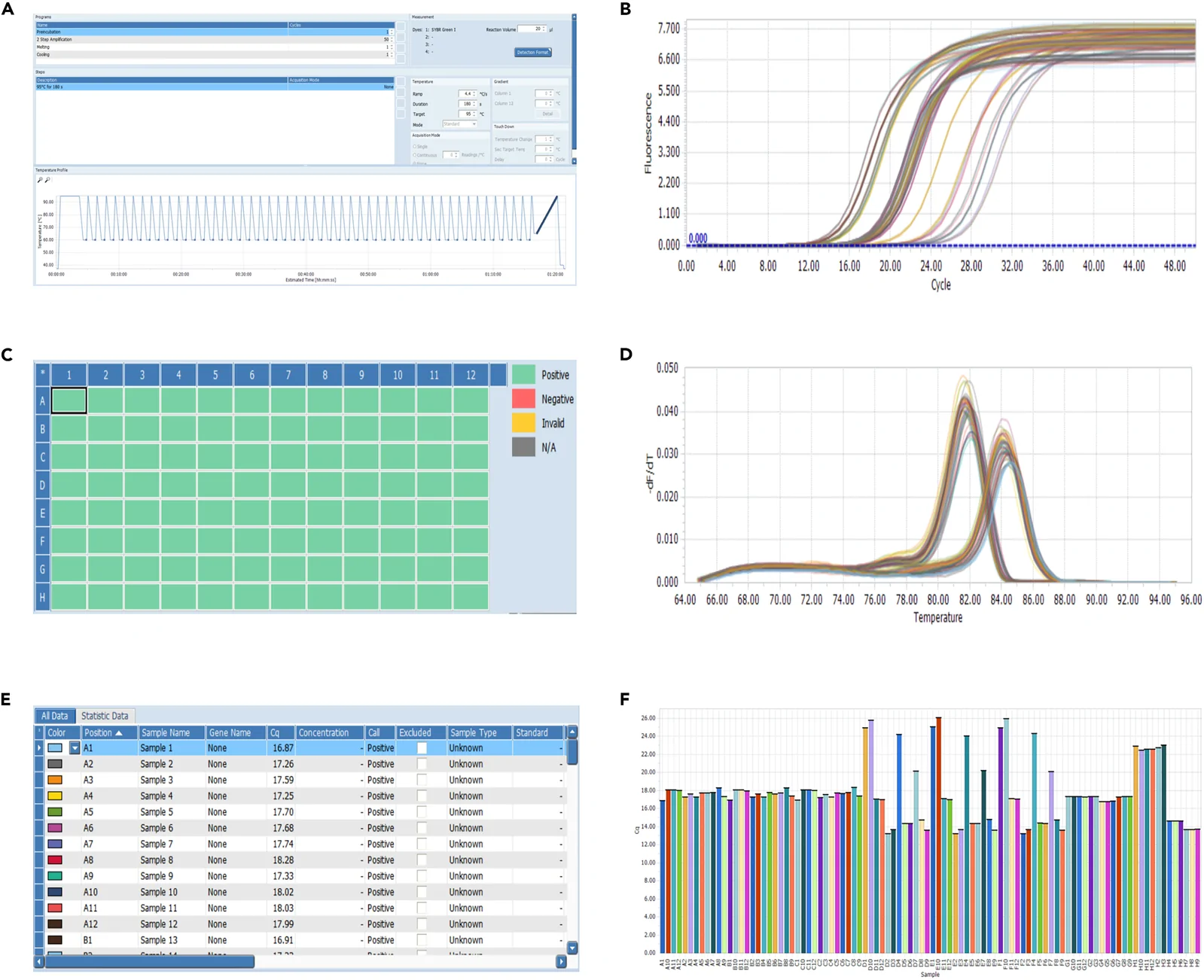
Quality verification of qPCR assay for mechanically wounded tomato samples (A) Screenshot of qPCR instrument setting interface and fluorescence baseline preview. (B) Amplification curves of target genes from all cDNA samples, showing typical sigmoidal amplification profiles without abnormal drift. (C) 96-well plate layout heatmap; all reaction wells were marked as positive amplification (green), with no negative or invalid wells. (D) Melting curves of amplified products, presenting single sharp peaks to confirm specific primer amplification without non-specific bands or primer dimers. (E) Statistical data table displaying Ct values, sample labels and amplification qualification status for each reaction. (F) Histogram of Ct value distribution across all tested SAM samples from mechanical damage treatment groups.
    ***Note:*** The annealing temperature should be empirically determined and optimized for each specific primer set.
    ***Note:*** Alternative qPCR platforms, such as TaqMan probe-based assays, may also be used, provided they are validated for the target genes and produce specific, reproducible amplification.
    **CRITICAL:** Melt curve analysis is required after qPCR amplification to confirm amplicon specificity. Heat from 60°C to 95°C at a rate of 0.3°C/s, with continuous fluorescence acquisition.
9. Data analysis:
  a. Normalize the expression levels of the target genes against those of tomato ACTIN2 (Figure 6).

**Figure 6:**
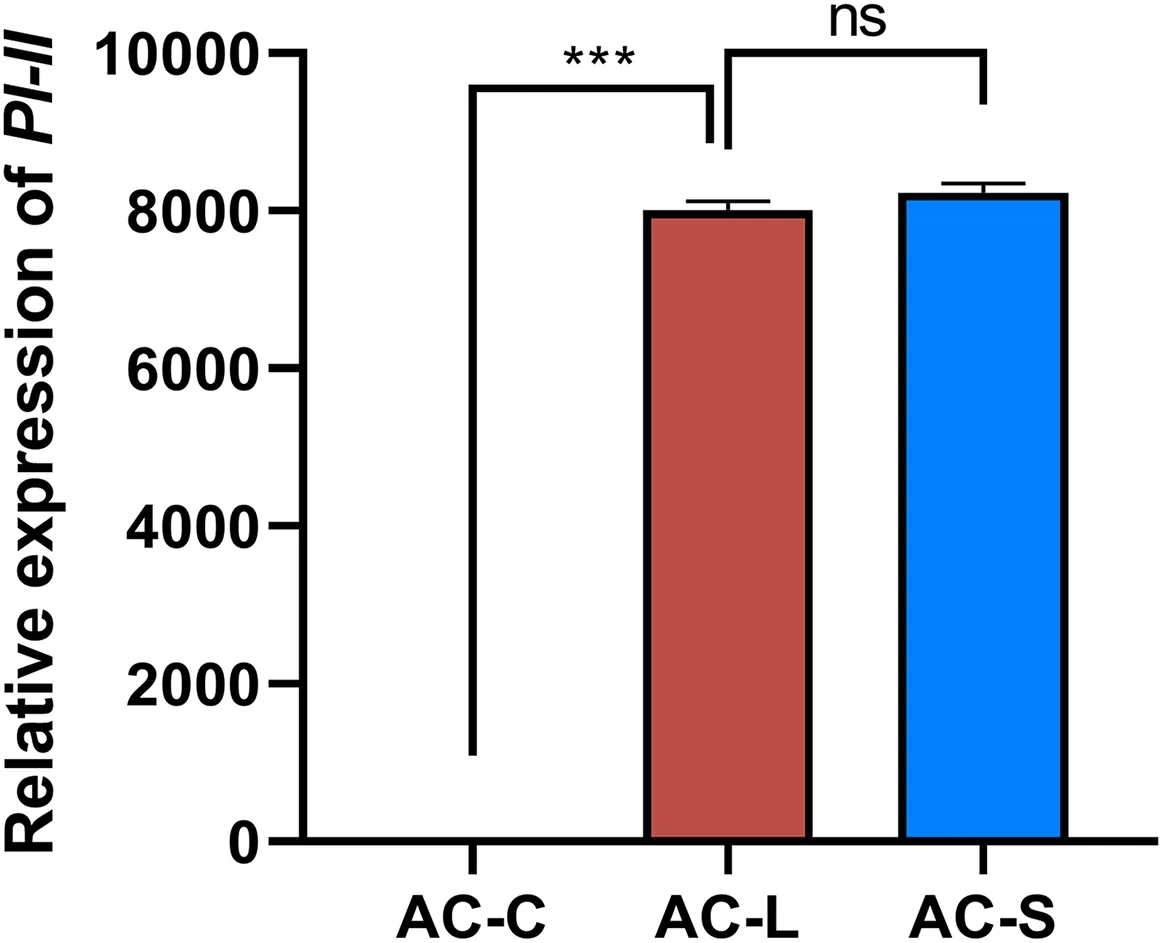
Representative qPCR result using this protocol Relative *PI-II* transcript levels in control, wounded local leaf, and systemic leaf of tomato plants at 12 h post wounding. Error bars represent SEM. One-way ANOVA followed by Tukey’s post-hoc multiple comparison test was used to calculate *p*-value.
  b. Select appropriate statistical tests to determine the significance of differences according to the experimental design.

***Note:*** Define significance standards: ∗*P*<0.05; ∗∗*P*<0.01; ∗∗∗*P*<0.001; ns means no significant difference. A two-tailed Student’s *t*-test is used for pairwise comparisons between two groups. For datasets with multiple genotypes, tissue types, or time points, ANOVA followed by post-hoc multiple comparison tests are recommended.

## Expected outcomes

With this comprehensive approach, it is possible to standardize the molecular analysis of systemic resistance responses of tomatoes, and then use it for the study of damage-induced systemic signal transduction, transcriptional regulation and physiological responses to activation.

In the resting state without any damage, the tomato seedlings do not detect the expression of resistance genes themselves. However, when the tomato seedlings suffer mechanical damage, the induction level of resistance genes represented by *PI-II* will rapidly increase and reach its peak 12 h after wounding. The induction factor of the *PI-II* gene can reach 8,000 times.

By establishing a controllable mechanical damage system, researchers were able to develop a time-related resistance response research process, including the specific location of mechanical wounding on plant seedlings, the sampling time points after wounding, and the sampling method for the leaves. These data are helpful in clarifying the gene functions and the systemic impact of local mechanical damage on the plant defense pathways.

RNA from wounded and systemic leaves can give high quality nucleic acids for transcriptional analysis. Reverse transcription qPCR can elucidate gene expression profiles related to wound-induced systemic responses, including upregulation of systemin, markers of JA pathways, etc. The present protocol can be used to analyze regulatory networks for local damage signals and activation of systemic defense responses.

In summary, this protocol provides a systematic technical method for exploring the molecular mechanism of a wound-triggered systemic wound response in tomato and enables comprehensive assessments of plant resistance, at the phenotypic, transcriptional and physiological levels. Moreover, the experimental procedures described here can be readily applied to other plant species with conserved systemic defense pathways, providing a reliable tool for future studies on plant stress memory and environmental adaptation mechanisms.

## Quantification and statistical analysis

All raw Ct values exported from the LightCycler 480 system were used for relative gene expression quantification. Only Ct values ranging from 18 to 35 with single sharp melting peaks were retained for downstream analysis; samples with abnormal melting curves, Ct > 35 or obvious technical replicate deviations > 1.0 Ct were excluded to avoid unreliable quantification. Relative transcript levels of target genes (*PI-II*) were normalized to the reference gene *ACTIN2* using the 2^−ΔΔCt^ method. ΔCt = Ct(target gene) − Ct(ACTIN2), and ΔΔCt = ΔCt(treated sample) − ΔCt(untreated control sample). Three independent biological replicates with two technical replicates per sample were prepared for all treatments.

Statistical comparisons between mechanical wounding groups and untreated control groups were performed using Student’s *t* test for two-group comparisons in GraphPad Prism. *P* < 0.05 was defined as the threshold for statistically significant differences. All relative expression data were presented as mean ± standard error of the mean (SEM). Expression graphs were plotted using GraphPad Prism. ImageJ v1.54f was used to quantify leaf wound area if phenotypic quantification was required.

## Limitations

Although systemic resistance phenotypes are judged based on marker gene expression and leaf physiological status, wound-triggered defense intensity varies greatly among tomato genotypes, seedling ages and growing surroundings. Genetic background and cultivation conditions alter the magnitude of systemic signaling upon mechanical injury. Thus, optimal sampling time points require experimental proof when testing different varieties of tomatoes or culture media.

This protocol relies on consistent artificial culture condition to allow repeatable mechanical wounding treatment. Under open-field growing conditions, sudden change of temperature, illumination and soil nutrition in disturbance, it also hinders wound-triggered systemic response. In this sense, the method works best under controlled lab condition and cannot completely represent the complex natural field environment.

Besides, total RNA extracted from distal systemic leaves is ideal for RT-qPCR detection of transcription of the defense genes, but bulk leaf sample integrates multiple cell types together. Bulk leaf RNA cannot resolve cell-type specific gene expression, which, furthermore, limits further studies on the single cell resolution of systemic defense signalling.

## Troubleshooting

### Problem 1

Uneven seed germination resulted in conspicuous difference in seedling size and growth stage (Step 1).

### Potential solution

• Before sowing, screen seeds of similar plumpness, equally large protruding radicle length; discard excessively large and shriveled seeds or no germinated seeds, which narrows the initial individual variance.
• Overspread evenly the seeds in each plug tray to balance the micro-environment variation of each plug such as light strength, soil moisture, temperature gradient in incubator.
• Maintain consistent culturing parameters (photoperiod, humidity and nutrient supply) throughout the whole germination and seedling growth period.
• If seedling heterogeneity still exists before treatment, sort seedlings by plant height and leaf number, only retain plants with uniform growth phenotype for subsequent mechanical damage assays.

### Problem 2

Seedlings suffer unintended abiotic stress during cultivation (Step 2).

### Potential solution

• Avoid overwatering or drought stress; keep substrate humidity stable throughout seedling growth.
• Strictly maintain fixed growth-chamber temperature, light intensity and air humidity as set parameters.
• Prevent mechanical bumping, insect contamination or sudden environment change before wounding treatment. Avoid unnecessary mechanical disturbances such as touching or shaking seedlings, direct airflow from growth chamber air conditioning, and water spray onto leaves, as these stimuli can trigger unintended changes in gene expression.
• Discard short weak, yellowed seedlings and stressed seedlings and choose the healthy strong seedlings for later experiment.

### Problem 3

Different degree of wound for each leaf of different individuals; different induction levels of defense gene expression (Step 3).

### Potential solution

• Choose tomato seedlings of two-leaf one-heart plant age with the same size and growing status before experiment to reduce the difference among individuals.
• Fully sterilize hemostatic forceps with 75% ethanol and wait for complete air-drying to avoid chemical injury interfering with wound response.
• Fix clamping position and clamping range on the first true leaf for every seedling to keep identical wounded area.
• Complete all wounding operations continuously within a short time window to avoid fluctuation of ambient temperature affecting physiological responses.

### Problem 4

RNA degradation or low total RNA yield after leaf grinding and extraction (Step 5–6).

### Potential solution

• Keep samples immersed in sufficient liquid nitrogen throughout grinding and avoid partial thawing of leaf powder.
• Grind leaf tissues into fine powder without bulk tissue pieces visible; insufficient grinding will reduce nucleic acid release yield.
• Transfer ground powder into the RNase free collection tubes, as quickly as possible, to reduce exposure time of samples at room temperature.
• Carefully follow the centrifuging speed and time; prevent cross-contamination of aqueous supernatant from protein layer during RNase-removal supernatant collection.

### Problem 5

Unsatisfactory RNA purity observed by NanoDrop (too low A260/A280, or too low A260/A230 ratio) (Step 6).

### Potential solution

• Avoid the contamination with residual phenol, ethanol or carbohydrate of the chloroform extraction process and washing steps of column.
• Dry the adsorption column completely after RW2 washing to remove residual ethanol from column before column elution.
• Use qualified RNase-free water for final RNA elution to avoid exogenous impurity pollution.

### Problem 6

Genomic DNA residue exists in final cDNA products after reverse transcription (Step7).

### Potential solution

• Strictly prepare gDNA removal system on ice and ensure accurate dosage of gDNA Eraser and matching buffer.
• Incubate 2 min at 42°C completely. Do not ever reduce reaction time for genomic DNA elimination.
• Prepare master mixes separately for gDNA digestion and RT setup to avoid mixing errors in reagents.

### Problem 7

Abnormal Ct value or qPCR detects a non-specific amplification (Step8).

### Potential solution

• If the template concentration is too high, dilute the cDNA appropriately with RNase-free water.
• Optimization of annealing temperature based on Tm value of the primers, to reduce non-specific binding.
• Run melting curve analysis after amplification; discard data with miscellaneous melting peaks.

### Problem 8

Large data fluctuation and poor repeatability of target gene relative expression (Step9).

### Potential solution

• Set more biological replicates and technical replicates for each treatment group to lower experimental error.
• Use internal reference of stable housekeeping gene *ACTIN2* for uniform data normalization.
• Avoid the repeated freeze-thaw cycles of reserved cDNA, which leads to uneven degradation of templates among batches.
• Use consistent and careful pipetting technique; pipetting errors are a major source of variability in qPCR assays.

## Resource availability

### Lead contact

Requests for further information, resources, and reagents should be directed to and will be fulfilled by the lead contact, Changbao Li (lichangbao@nercv.org).

### Technical contact

Technical questions on executing this protocol should be directed to and will be answered by the technical contact, Qinyang Wang (qywanggenetics@163.com).

### Materials availability

This study did not generate any new materials or reagents.

### Data and code availability

This study did not generate any new data or code.

## Acknowledgments

This study was supported by grants from the 10.13039/501100001809National Natural Science Foundation of China (no. 32573048).

## Author contributions

Changbao Li, Chuanyou Li, and L.D. designed and supervised the project. Q.W., M.Z., and G.Y. performed the experiments, analyzed the data, and prepared the figures. Q.W. and Changbao Li wrote and revised the manuscript. All authors have read and approved the final manuscript.

## Declaration of interests

The authors declare no competing interests.

## References

[1] Zhou K., Wu F., Deng L., Xiao Y., Yang W., Zhao J., Wang Q., Chang Z., Zhai H., Sun C. (2025). Antagonistic systemin receptors integrate the activation and attenuation of systemic wound signaling in tomato. Dev. Cell.

[2] Ryan C.A. (1974). Assay and biochemical properties of the proteinase inhibitor-inducing factor, a wound hormone. Plant Physiol..

[3] Wang L., Wang Y., Felix G. (2019). Peptide feeding and mechanical wounding for tomato seedlings. Bio. Protoc..

[4] Li L., Li C., Lee G.I., Howe G.A. (2002). Distinct roles for jasmonate synthesis and action in the systemic wound response of tomato. Proc. Natl. Acad. Sci. USA.

[5] Yang T., Deng L., Wang Q., Sun C., Ali M., Wu F., Zhai H., Xu Q., Xin P., Cheng S. (2024). Tomato CYP94C1 inactivates bioactive JA-Ile to attenuate jasmonate-mediated defense during fruit ripening. Mol. Plant.

[6] Yang W., Zhai H., Wu F., Deng L., Chao Y., Meng X., Chen Q., Liu C., Bie X., Sun C. (2024). Peptide REF1 is a local wound signal promoting plant regeneration. Cell.

[7] Schneider C.A., Rasband W.S., Eliceiri K.W. (2012). NIH Image to ImageJ: 25 years of image analysis. Nat. Methods.

[8] Jiang S., Li H., Zhang L., Mu W., Zhang Y., Chen T., Wu J., Tang H., Zheng S., Liu Y. (2025). Generic Diagramming Platform (GDP): a comprehensive database of high-quality biomedical graphics. Nucleic Acids Res..

